# EnderScope: a low-cost 3D printer-based scanning microscope for microplastic detection

**DOI:** 10.1098/rsta.2023.0214

**Published:** 2024-06-03

**Authors:** Niamh Burke, Gesine Müller, Vittorio Saggiomo, Amy Ruth Hassett, Jérôme Mutterer, Patrick Ó Súilleabháin, Daniel Zakharov, Donal Healy, Emmanuel G. Reynaud, Mark Pickering

**Affiliations:** ^1^ School of Medicine, University College Dublin, Dublin, Ireland; ^2^ Multiscale Biology, Johann-Friedrich-Blumenbach Institut für Zoologie und Anthropologie, Georg-August-Universität Göttingen, Gottingen, Niedersachsen, Germany; ^3^ Department of BioNanoTechnology, Wageningen University and Research, Wageningen, Gelderland, The Netherlands; ^4^ Institut de Biologie Moléculaire des Plantes, CNRS & Strasbourg University, Strasbourg, France; ^5^ Department of Psychology and Neuroscience, Boston College, Chestnut Hill, PA, USA; ^6^ School of Biology & Environmental Science, University College Dublin, Dublin, Ireland; ^7^ UCD Centre for Biomedical Engineering, University College Dublin, Dublin, Ireland

**Keywords:** open-hardware, low-cost, accessible microscopy, 3D printing, microplastic pollution

## Abstract

Low-cost and scalable technologies that allow people to measure microplastics in their local environment could facilitate a greater understanding of the global problem of marine microplastic pollution. A typical way to measure marine microplastic pollution involves imaging filtered seawater samples stained with a fluorescent dye to aid in the detection of microplastics. Although traditional fluorescence microscopy allows these particles to be manually counted and detected, this is a resource- and labour-intensive task. Here, we describe a novel, low-cost microscope for automated scanning and detection of microplastics in filtered seawater samples—the EnderScope. This microscope is based on the mechanics of a low-cost 3D printer (Creality Ender 3). The hotend of the printer is replaced with an optics module, allowing for the reliable and calibrated motion system of the 3D printer to be used for automated scanning over a large area (>20 × 20 cm). The EnderScope is capable of both reflected light and fluorescence imaging. In both configurations, we aimed to make the design as simple and cost-effective as possible, for example, by using low-cost LEDs for illumination and lighting gels as emission filters. We believe this tool is a cost-effective solution for microplastic measurement.

This article is part of the Theo Murphy meeting issue 'Open, reproducible hardware for microscopy'.

## 1. Introduction

As imaging tools have become more specialized in recent years, they have also become more expensive, placing these technologies beyond the reach of many of those who could benefit from them (e.g. researchers in low- and middle-income countries and those outside of the traditional research laboratory environment). In parallel to these technological developments, recent years have also seen considerable growth in a community of imaging scientists committed to open and accessible microscopy, allowing for local solutions to global-scale issues [1]. Here, we present an open-source imaging tool specifically designed to tackle the problem of marine microplastic (MP) pollution.

MPs are defined as small plastic particles <5 mm in size [2]. Due to the way in which MPs are transported in the environment, many will end up in the sea, where they are called marine MPs [3]. While marine MP pollution is a global problem, our understanding of the extent of this problem, and therefore our ability to assess the effectiveness of any mitigation strategies, remains limited. According to the UN Sustainable Development Goals (specifically target 14.1), there is ‘no internationally established methodology or standards [yet] available’ to measure floating plastic debris such as marine MPs [4].

The current dominant approaches to measuring marine MPs involve first digesting the organic material in the samples, for example, using strong acids, bases, oxidizing agents or through enzymatic digestion [5]. The digested sample is then filtered and techniques such as Fourier transform infrared spectroscopy (FTIR) or Raman spectroscopy are used to detect the filtered plastic particles [6]. However, both Raman and FTIR spectroscopy require instruments costing tens of thousands of euros.

An alternative method of measuring marine MPs is available at a lower cost. The sample can be stained with the fluorescent dye, Nile Red, which binds to MPs making them measurable using traditional fluorescence microscopy [7,8]. While lower in instrumental cost than spectroscopic methods, this approach is labour-intensive and time-consuming. An automated and low-cost fluorescence microscope would not only make imaging MPs more efficient but also accessible to a wider range of users.

**Figure 1.**
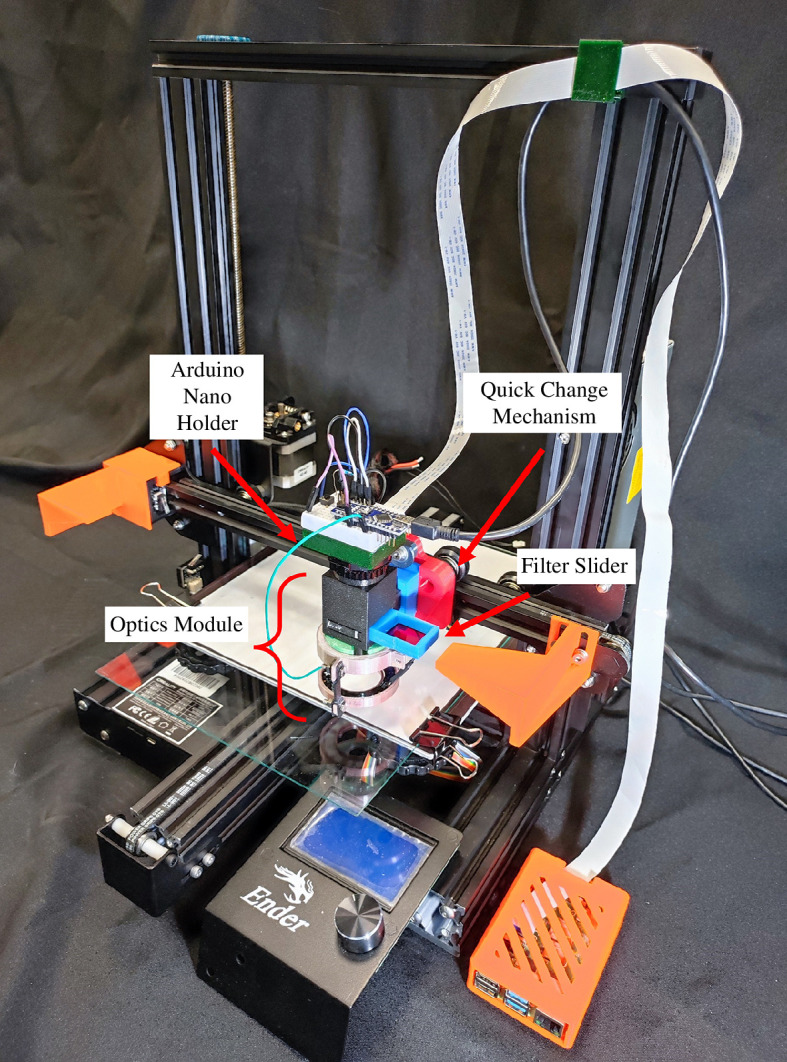
The EnderScope. The optics module (see figure 2 for more detail) consists of a 3D-printed ‘Lens Tube’, Raspberry Pi High Quality C-Mount Camera, lighting gel emission filter mounted in a 3D-printed ‘FilterSlider’, NeoPixel LED ring mounted in a 3D-printed ‘NeoPixel Mount’, an Arduino Nano wired to a pushbutton for control of the NeoPixel ring, a low-cost generic brand 4× finite conjugate objective lens and a Raspberry Pi single-board computer. The optics module is mounted to the *x*-axis carriage of a Creality Ender 3 Pro 3D printer using a 3D-printed quick change mechanism (figure 3).

We have designed a low-cost microscope for automated scanning and detection of MPs in filtered seawater samples—the EnderScope (figure 1). This microscope is based on the mechanics of a low-cost, open-source 3D printer, the Creality Ender 3 Pro (Shenzhen Creality 3D; [9]). Using the reliable and calibrated motion system of the 3D printer, it is possible to automate the scanning of a large filtered sample, as large as the print bed (>20 × 20 cm). We aimed to simplify and reduce the cost of this tool where possible, prioritizing accessibility, using 3D printing of optomechanical components or low-cost commercial components where possible.

**Figure 2.**
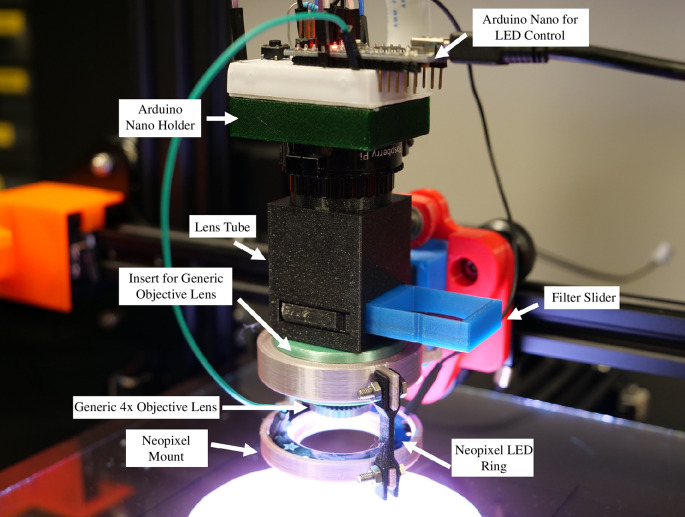
The EnderScope Optics Module. A Raspberry Pi High Quality C-Mount Camera is connected to one end of a 3D-printed ‘Lens Tube’. A 4× finite conjugate objective lens is connected to the opposite end of the lens tube. Two red lighting gels are layered on top of each other and inserted in a 3D-printed ‘Filter Slider’. The ‘Filter Slider’ is inserted into the ‘Lens Tube’ and can be moved back and forth via a 3D-printed flexure mechanism. A 3D-printed insert (in green) is positioned around the 4× objective lens, allowing for the ‘NeoPixel Mount’ (in pink) to be easily positioned around the objective lens. A NeoPixel ring is placed in the ‘NeoPixel Mount’. In dark green, a 3D-printed ‘Arduino Nano Holder’ slides onto the Raspberry Pi camera board. A 170 pin bread board with adhesive backing is stuck on top of the Arduino Nano Holder. The NeoPixel ring is wired up to the Arduino and push button as shown in the provided assembly instructions (electronic supplementary material, file 1).

**Figure 3.**
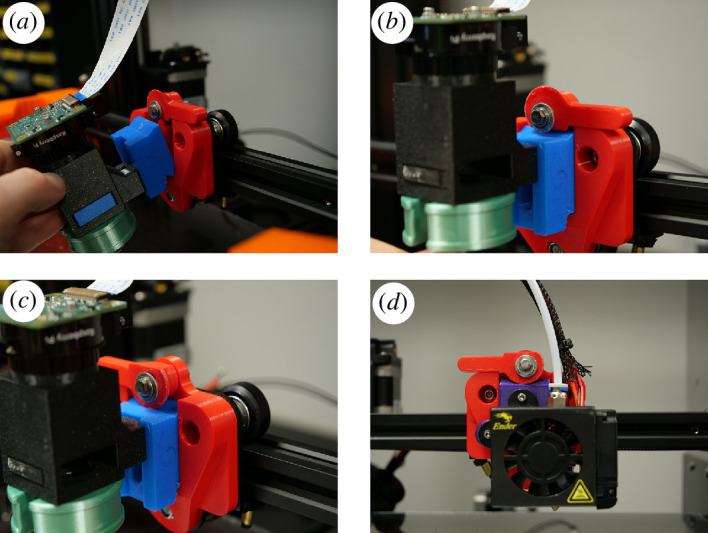
The EnderScope Quick Change Mechanism. The Quick Change Mechanism on the EnderScope was designed to allow for easy swapping between the EnderScope optics module and the hotend of the Ender 3 Pro. (*a*) and (*b*) show the ‘EnderScope Tool Holder’ from the EnderScope Optics module being inserted into the ‘Tool Holder Base’. (*c*) The ‘Lever’ is then rotated so that the curved side of the lever is clamping the EnderScope ToolHolder in place. (*d*) With the hotend of the Ender 3 Pro attached to the ‘Ender Hotend ToolHolder’, the hotend can be mounted to the ‘Tool Holder Base’ in the same way.

## 2. EnderScope hardware and software description

### 2.1. Design principles

In developing the EnderScope, we made a deliberate effort to prioritize two core design principles: the instrument should be low cost, and it should be simplified both in terms of optomechanical design and operation. Therefore, rather than trying to design the most capable instrument possible, we chose to design the simplest instrument that was still capable of meeting the minimum function of detecting MPs. The final design consists of a largely 3D-printed light microscope, capable of fluorescence and white light imaging, that reversibly replaces the extruder assembly on the *x*-axis carriage of an Ender 3 Pro 3D printer.

### 2.2. Hardware

#### 2.2.1. 3D modelling and printing

All 3D printable parts were designed in Tinkercad (Autodesk), with the exception of the RMS threads included in the ‘LensTube’ design, which were designed using Fusion 360 (Autodesk) using the thread creation tool. All design files are provided in the editable standard for the exchange of product data (STEP) format and in static stereolithography (STL) format as is considered best practice [10].

All 3D printable parts were printed in polylactic acid (PLA) using an Original Prusa i3 MK3S+ and tested using both the Ender 3 Pro and Ender 2 Pro. All parts are printed with a 0.2 mm layer height and no supports (except for the optional filter changer).

All design files and assembly instructions are available on the EnderScope Github repository: https://github.com/Pickering-Lab/EnderScope and have released the designs and software under an open-source licence (Hardware: CERN-OHL-P, Software: MIT, Documentation: CC-BY-4.0).

#### 2.2.2. Low in cost: building a simple fluorescence microscope

##### 2.2.2.1. Illumination source

Illumination for both fluorescence and reflected ‘white light’ imaging is provided by a low-cost NeoPixel LED ring mounted on the objective lens (NeoPixel ring, 16× RGB LED, 16× WS2812). The green LEDs in the RGB array, which can be activated independently, overlap with the excitation wavelength of Nile Red (excitation peak 543–553 nm; see electronic supplementary material, figure S1, for NeoPixel emission spectra) and matched activation of all three pixels in the array provides ‘white’ light illumination. The LED ring is mounted in a 3D-printed holder that is clipped around a 3D-printed adapter that attaches around the objective lens. We have designed adapter inserts to accommodate objective lenses with varying diameters, such as a Nikon Plan 4×, Olympus PLN 4× and more affordable generic lenses. This allows the same illumination system to be used with a range of objectives, and attaching to the objective allows the height of the LED array to be adjusted to match the working distance of the objective to provide even illumination. The NeoPixel ring is controlled by an Arduino Nano microcontroller (ATmega328) as shown in figure 2 with a pushbutton allowing switching between white (reflectance) and green (fluorescence) illumination modes.

The microscope control and acquisition system uses a Raspberry Pi single-board computer. While the Raspberry Pi GPIO is capable of controlling the LED array, we chose instead to use a separate microcontroller for this purpose, as the Arduino is more robust than the Raspberry Pi, which can be damaged by voltage spikes or short circuits resulting from incorrect wiring or operation.

##### 2.2.2.2. Objective lens

We used a generic, low-cost, RMS threaded 4× finite conjugate objective lens (4 × 185 Biological Microscope, Standard Plan Achromatic Objective Lens, NA = 0.1) available for approximately €8 at the time of writing. We have shortened the tube length from the standard 160 mm to approximately 60 mm for ease of use. While this decreases the effective magnification and can introduce distortions such as vignetting, it allows for the optics module to be more portable and increases the working distance to approximately 3 cm with a corresponding increase in depth of field (DOF). The 3D-printed lens tube features internal RMS threads on one end, for attachment of the objective lens, and external C-Mount threads for attachment of the C-Mount Raspberry Pi High Quality Camera on the opposite end.

##### 2.2.2.3. Emission filter

The fluorescence imaging mode of the EnderScope requires an emission filter in the detection path. Instead of using a traditional glass emission filter, we have opted to use a thin plastic film filter (sold commercially as lighting gels). Lighting gels are thin sheets of coloured plastic commonly used in the entertainment industry or photography to change the colour output of a light. Two small squares are cut from a red lighting gel (Neewer 35PCS Universal Photography Speedlite 47 × 77 mm Square Full Color Balance Gel Filter Kit) and placed in the 3D-printed filter slider layered on top of each other (figure 2). The filter slider is placed in the 3D-printed lens tube and can be aligned in or out of the light path with a flexure mechanism in the lens tube.

##### 2.2.2.4. Camera

We opted for a Raspberry Pi High Quality C-Mount Camera as it is a fraction of the price of traditional cameras for microscopy. Compared with its predecessors (the Raspberry Pi Camera Module v.1 and v.2), the High Quality camera has a larger sensor (7.9 mm versus 4.6 mm diagonal), with more pixels (4056 × 3040 versus 3280 × 2464), larger pixels (1.55 × 1.55 µm versus 1.12 × 1.12 µm) and more sensitive pixels (owing to the larger pixel area), which is ideal for fluorescence and low light imaging. The C-Mount threads on the ‘LensTube’ part will allow for any C-Mount camera to be used. The only consideration to keep in mind is the weight of the camera. Many traditional microscopy cameras are quite heavy. We would recommend smaller machine vision cameras in place of these, such as those used in Courtney *et al*. [11], which are typically less expensive and smaller than conventional microscopy cameras. If using the Raspberry Pi High Quality Camera (or any other Raspberry Pi Camera), a CSI-USB adapter could be used to allow for laptop operation.

### 2.2.3. Easy to build and simple design: retaining the printer’s 3D printing capabilities

Several designs that repurpose 3D printers for scientific instruments have been published. However, these often require disassembly or extensive modification, which necessarily requires the loss of 3D printing functionality. For example, the syringe pump system developed by Baas and Saggiomo [12], which uses the components of an Ender 3 3D printer, and the Incubot, a live cell automated incubator microscope designed by Merces *et al*. [13] both require effectively sacrificing a 3D printer in their construction.

The HistoEnder is another instrument based on 3D printer hardware (specifically a histology autostainer based on an Ender 3D printer [14]). However, the HistoEnder leaves the 3D printer fully intact and retains its 3D printing capabilities. This dual-use design effectively increases the cost-effectiveness of the instrument.

When designing the EnderScope, we decided to adopt a similar approach to the HistoEnder. This is achieved through a 3D-printed quick change mechanism, allowing the user to easily swap between the hotend of the original 3D printer and the optics module (figure 3). This design was adapted from ‘Quick tool change for Creality CR-10/Ender series’ by Jón Schone (ProperPrinting) on Thingiverse [15], CC-BY-SA 3.0. The quick change mechanism on the EnderScope facilitates the dual-use design and also allows the 3D printer to be used to print the microscope components needed during the EnderScope construction. Warping or melting of the 3D-printed ‘Ender Hotend Tool Holder’ should not occur when the EnderScope is used as a 3D printer. This is because this part is bolted to the cold end of the printer tool head, which should not get hot enough to melt the 3D print. If warping is observed over time, the part can be printed in a different material with a higher melting temperature, e.g. acrylonitrile styrene acrylate (ASA), or replaced when needed.

### 2.3. Software and control

The EnderScope is connected to a Raspberry Pi (Model 4 B Rev 1.1 4 GB) via a serial over USB connection, allowing G-code commands (which control the motion systems on a 3D printer) to be sent from the Raspberry Pi to the Ender 3D printer using a Python script. This approach requires no modification of the printer firmware, with all customization and control running from the Raspberry Pi. While automation and control of the EnderScope can be customized according to the user’s needs, we have provided example scripts (electronic supplementary material, file 1) to demonstrate how the EnderScope can be controlled via Python, allowing new users to use the instrument without the need to programme custom control systems.

#### 2.3.1. Graphical user interface

**Figure 4.**
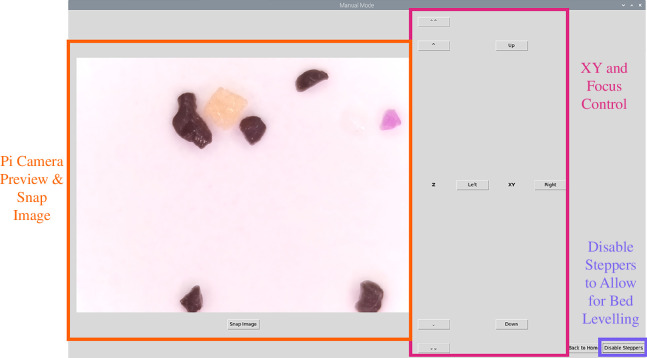
Manual mode graphical user interface (GUI). This GUI includes buttons that, when pressed, send G-code commands to the Ender 3D printer to move in X, Y or Z. The GUI also includes a Raspberry Pi Camera preview window and a button that, when pressed, will save an image. Finally, the GUI contains a button that will disable the stepper motors on the 3D printer. Once disabled, the user can manually move the optics module to each corner of the Enderprint bed to perform bed levelling as per EnderScope assembly instructions (electronic supplementary material, file 1).

A basic user interface was designed using the Python library, Tkinter [16]. This provides buttons for X, Y and Z motion of the microscope, a live preview image with a button to save this image, and a button to disable steppers to allow for manual bed levelling (figure 4). Bed levelling should be performed before imaging, which removes the necessity for autofocus controls during automated data acquisition. Moving the microscope to each of the four corners of the bed and manually adjusting the focus with the bed levelling screws will give a level plane of focus aligned to the xy plane of movement. We have provided a protocol for optical bed levelling along with the assembly instructions for the EnderScope (electronic supplementary material, file 1).

#### 2.3.2. Snake scan

For automated image acquisition, we created a Python script that moves the printer in a snake-like scan pattern covering a large area, acquiring images in each position. Overlap between images can be controlled if stitching is required.

## 3. Validation

### 3.1. Optical validation

#### 3.1.1. Resolution estimation

While resolution can be measured using several methods including the Abbe diffraction formula, Rayleigh criterion or full-width half maximum calculated from the point spread function, we have chosen to estimate the resolution of our instrument using the slanted edge modulation transfer function (MTF) method. Unlike the previously mentioned methods, MTF is a function of the entire optical system. MTF must therefore be determined each time an element in the optical system is changed (e.g. changing the objective lens or inserting fluorescence emission filters in the light path). Therefore, MTF was used to quantify the resolution of our system in both reflected light and fluorescence modes. Slanted edge MTF was measured as in previous optical characterizations of custom microscopy systems (specifically [11,13]).

In fluorescence mode, the modulation drops off at a lower resolution compared to reflected light mode (electronic supplementary material, figure S2*a,b*), reflecting the impact of the gel filter in the optical path. In both reflected light and fluorescence modes, the modulation of the images drops off well before we reach the maximum resolution the system can operate at (approx. 300 lp/mm). As our resolution was optically limited rather than sensor limited, we opted for 4× binning during image acquisition, reducing image dimensions to 1014 × 760 from the original 4056 × 3040 resolution.

Binning the images 4× , we compared the MTF profiles of the low-cost, generic brand 4× finite conjugate lens used in the EnderScope design to a Nikon CF N Plan Achromat 4×/0.13 objective lens (electronic supplementary material, figure S2*c,d*). The resolution profile of both objectives is similar, implying little justification for using the more expensive Nikon objective in the system compared with the significantly lower-cost generic objective.

#### 3.1.2. Depth of field

Using the snake scan script, the EnderScope can be used to image over a large area in a single plane. While the script can be modified to include autofocus capabilities, we have avoided this to keep the automation as simple as possible. In order to restrict imaging to one plane, we must ensure that the DOF of our objective lens is large enough to capture the size of a typical MP. To assess the DOF of the generic lens, a small 3D-printed wedge shape was imaged using the EnderScope in reflected light mode. The wedge was printed in PLA at 0.05 mm layer height, meaning each layer of the wedge is spaced 0.05 mm apart in Z. In the image acquired (electronic supplementary material, figure S3) 10 layers of the wedge are in focus, meaning the DOF is approximately 0.5 mm. This means we can capture MPs up to 0.5 mm tall in Z without the need for autofocus.

#### 3.1.3. Relative illumination

To measure the relative illumination (RI) of an image taken using the EnderScope, a USAF 1951 resolution test target (Thorlabs, R3L1S4P) was raised on a platform approximately 40 mm above a sheet of white paper. The paper provides a white background for the image. The target is raised above the paper to ensure a consistent and clean background, minimizing the influence of any paper imperfections or dust particles on the RI measurement. The EnderScope was focused on a group of lines on the target. Keeping the Z position the same, an image was taken of a blank area of the target. The NeoPixel ring was set to white light at maximum intensity. The mean grey value across a diagonal line on the sensor was measured in Fiji [17]. This was then expressed as a percentage of the maximum grey value measured along this diagonal on the sensor and plotted against the distance in pixels across the sensor.

We see relatively even illumination across the diagonal of the camera sensor with no signs of vignetting effects in our image (electronic supplementary material, figure S4).

#### 3.1.4. Geometrical distortion and spherical aberration

Finally, the effects of distortion and spherical aberration on our system were measured. When shortening the tube length of a finite conjugate objective lens, as we have done in the EnderScope, it can worsen the effects of distortion and spherical aberration.

To measure the effects of distortion on our system the Distortion Correction plugin was used in Fiji [18]. A 3 × 3 grid of images was taken of a sample with high contrast and 50% overlap in X and Y. Our chosen sample was a printed picture on card as this has enough contrast and structure to ensure the plugin can detect correspondence points between images.

While we did detect some distortion in the images towards the edges (electronic supplementary material, figure S5), the overall distortion is low, with features at the edge of the image deviating from their expected positions by <2 pixels. The effects of spherical aberration were measured from an image taken of Thorlabs distortion target (R2L2S3P3) acquired using the EnderScope with the generic objective lens. In Fiji, the pixel intensity was measured along a line along the *x*-axis of the image, starting from midway through the *y*-axis of the image. It was ensured that this line was passing through the approximate centre of each dot it encountered. We see a slight drop in sharpness at the edges of the image (electronic supplementary material, figure S6).

### 3.2. Mechanical validation

#### 3.2.1. Positional error

To assess the movement repeatability of the EnderScope, a series of repeated 6 mm movements back and forth (29 movements in each direction, 58 movements in total) was performed, with an image acquired after each movement. This was repeated for both *x*-axis and *y*-axis movements. By locating the centre of a dot in each image, we were able to determine the dimension repeatability of the EnderScope motion system; the dot should appear in the same location after moving away and then back again if the movement is repeatable. Using this approach, we determined the positional deviation falls within ±4 µm in the *x*-axis and *y*-axis when moving the optics assembly along the *x*-axis and *y*-axis (electronic supplementary material, figure S7).

### 3.3. Power consumption

As there is a growing desire for researchers to understand the environmental impacts of their work, we provide the power consumption data for the EnderScope. All components of the EnderScope (Raspberry Pi, Arduino Nano for LED, computer monitor) were plugged into a power strip. This power strip was then plugged into a plugin power meter (Multicomp Pro MP001186). It was found that the EnderScope consumes 44 W while scanning a sample (table 1). By comparison, a typical Tungsten-Halogen bulb, commonly used in transmitted light microscopy, consumes about 100 W.

**Table 1.** Power consumption of EnderScope during a scan. Power consumption of EnderScope components was measured using a Multicomp Pro MP001186 power meter. Creality 3D Ender-3 Pro, Arduino Nano microcontroller (ATmega328), NeoPixel ring, 16× RGB LED, 16× WS2812, Dell Flat Panel Monitor P2417H, Raspberry Pi Model 4 B Rev 1.1 4GB, Dell MS116t USB wired optical mouse, Raspberry Pi Keyboard (RPI-KYB).

Component	Power consumption (W)
Ender 3 Pro	23.3
Arduino Nano and NeoPixel Ring	1.8
Computer monitor	14.5
Raspberry Pi Model 4 B Rev 1.1 4 GB keyboard mouse	4.4
Total	44.0

### 3.4. Proof-of-concept experiments

#### 3.4.1. Test of reflected light and fluorescence imaging modes

To test whether the EnderScope can be used to image fluorescent and non-fluorescent samples, a mixture of fluorescent (REAL fluorescent orange PLA filament 1.75 mm) and non-fluorescent (Prusament Jet Black PLA 1.75 mm, Prusa Research) PLA was imaged using the EnderScope. A block of tree supports was generated using PrusaSlicer (version 2.6.1) and printed with each colour filament. The support blocks were cut into smaller pieces before being shredded into fine particles using a blender (Texet nutri-vault, BB-6088). These particles were filtered through a 0.6 mm mesh (JBL Artemio 4, Sieve combination). Particles were placed on a dampened filter paper (Whatman 602H1/2, 90 mm diameter, 10312642). The filter paper was not used to filter the samples but to provide a realistic background for imaging.

**Figure 5.**
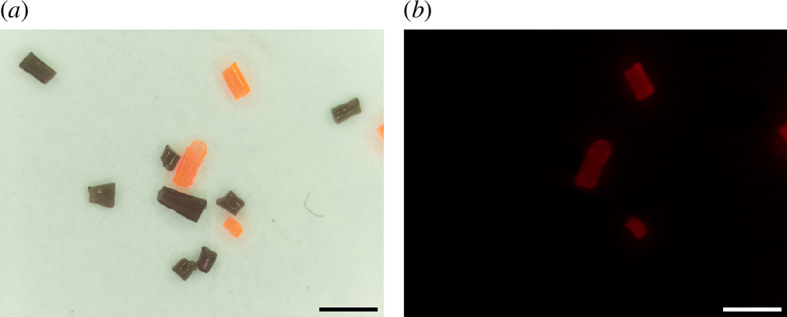
Reflected light and fluorescence imaging modes. Images of fluorescent (orange) and non-fluorescent (black) PLA plastic particles acquired using the EnderScope in reflected light (*a*) and (*b*) fluorescence modes. Scale = 1 mm.

As seen in figure 5, the EnderScope is capable of selectively detecting the orange fluorescent particles. This confirms that the green light illumination from the NeoPixel LED ring and the use of a lighting gel as an emission filter are sufficient for fluorescence imaging.

#### 3.4.2. Large-field scanning and particle analysis

**Figure 6.**
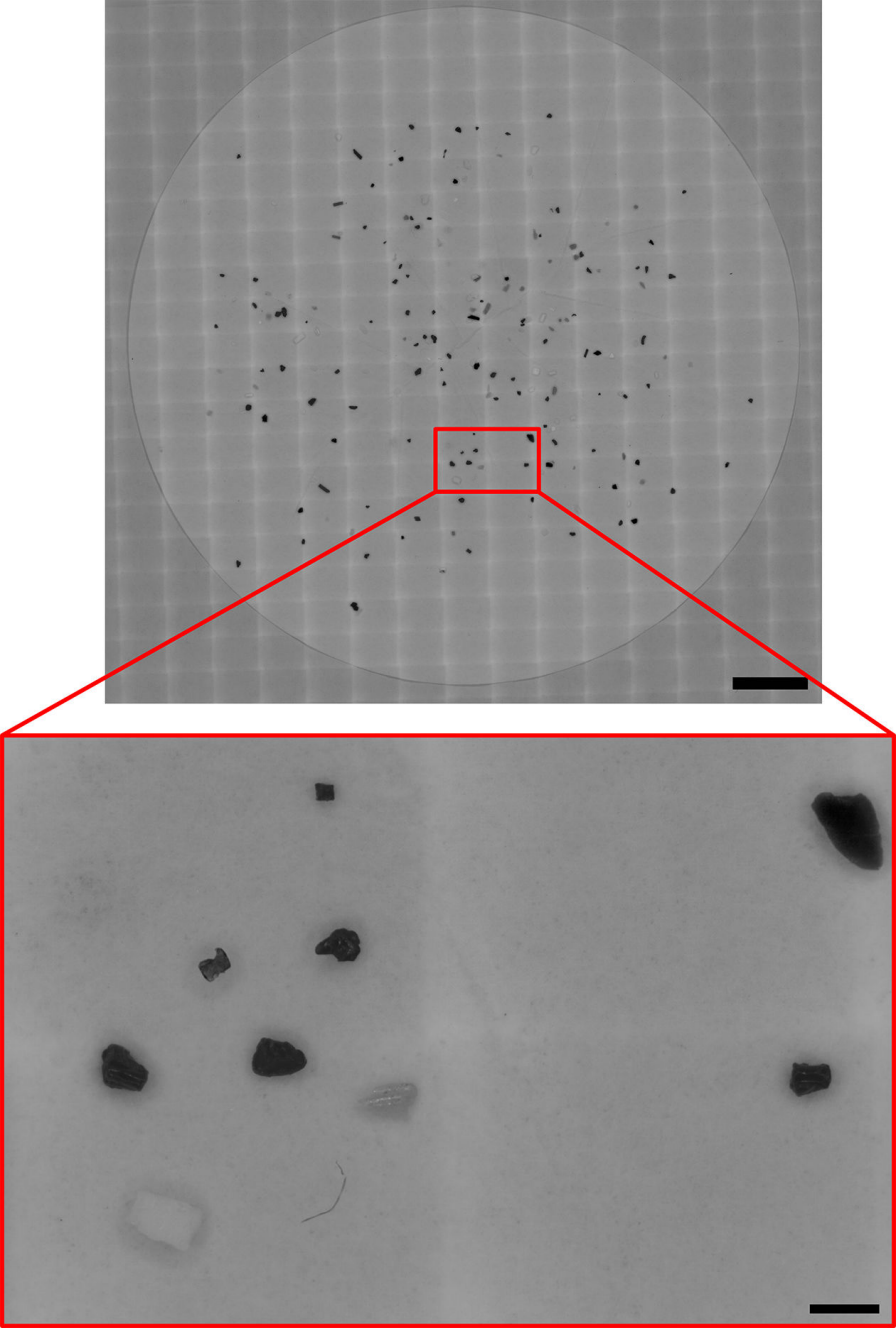
Composite image. Composite image of a filter paper containing shredded plastic particles (PETG, PLA, Nylon and ABS). This filter paper was imaged with a snake scan using the EnderScope in reflected light mode. These images were stitched using the BigSticher plugin in Fiji. The red callout box shows a selected region of interest on the filter. This selected region of four images shows plastics that cross image boundaries and are accurately stitched using BigStitcher. Scale for the composite image = 10 mm. Scale for the selected region of interest = 1 mm.

In this experiment, we tested the ability of the EnderScope to accurately scan over a large area and produce images that can be stitched into a large composite image. A mixture of shredded 3D printer filament (polyethylene terephthalate glycol (PETG), PLA, Nylon and acrylonitrile Butadiene Styrene (ABS)) was shredded further in a blender, filtered and placed on a dampened filter paper, as in §3.4.1. The entire filter paper was scanned using the SnakeScan.py script (parameters used in script are: x_win = 18, y_win = 25, x_step = 6.2, y_step = 4.5, exposure = 5000, wb.gain = 4, 1). Images were moved to a desktop computer and imported into Fiji. These images were flipped vertically and horizontally in Fiji, and the red channel was extracted. A large composite image was generated using the BigStitcher plugin in Fiji (figure 6) [19]. Not only does this composite image show we can successfully stitch images acquired using the EnderScope but it also provides assurance that the snake scan script is functioning as expected. A Gaussian blur filter was applied to the composite image (sigma = 12). The image was then thresholded (manual thresholding, default method), and particle detection was performed in Fiji (analyse > analyse particles). One hundred and thirty-eight particles were detected, and their size and circularity (an index of shape) were measured (electronic supplementary material, figure S8).

Detailed particle-level analysis requires the use of an additional computer to stitch composite images. Stitching these images on the Raspberry Pi through simple alignment using Python is difficult unless the camera can be aligned exactly to the motion axis of the printer and also requires perfect alignment of the printer’s *x-* and *y*-axes, which cannot be guaranteed. In keeping with the principle of making the device as simple and accessible as possible, we also created a simple Python script (electronic supplementary material, file 1) that can be run on the Raspberry Pi itself, which can run a simplified measurement. Here, the total plastic area in a sample is calculated by cropping each image to remove overlapping areas in the top *x*-axis and right *y*-axis. A threshold is applied to each image to differentiate between plastic particles and background. Thesholded areas are then summed to calculate the total plastic area in a sample. While this option does not provide information on the number of particles in a sample, their size or circularity, it provides a simple measure of the total plastic area in a sample and is a lightweight script that can be run on a Raspberry Pi.

Using this simple analysis on the same dataset used for the stitched particle analysis, the total area of plastics coverage was 50.56 mm², lower than the total of 71.74 mm² of plastics coverage detected using the more sophisticated analysis method. While the simplified method is less likely to give accurate results, this method may be adequate for some users depending on the use case (e.g. it may be sufficient for determining trends in MP distribution at a particular site over time).

#### 3.4.3. Particle detection in seawater sample

Next, we tested the EnderScope’s capabilities in a more authentic use case; detection of Nile Red-stained MPs from environmental samples.

A seawater sample was collected from Howth Harbour, Co. Dublin (53°23′35″N, 6°03′59″W) in October 2023. One litre of seawater was collected at high tide from the surface of the water. The seawater sample was treated with potassium hydroxide (KOH) [20–22]. KOH flakes (potassium hydroxide, reagent grade 90% flakes, Sigma Aldrich 484016-1KG) were added to the seawater sample to make a 10% w/v solution. The sample was left at 60°C overnight. The sample was then filtered through a stainless steel 125 µm sieve (Test Sieve, Endecotts Ltd., 667924). Material collected on the sieve was resuspended in distilled water and neutralized to a pH of approximately 6 with 1 M HCl. The resuspended sample was filtered through the metal sieve again, and the material on the sieve was covered with Nile Red (0.01 mg/ml in methanol, Nile Red for Microscopy, Sigma Aldrich, 72485-100MG) in the dark for 15 min. The stained plastics were both washed and resuspended with distilled water and filtered through a filter paper (Whatman 602H1/2, 90 mm diameter, 10312642). The filter paper was left to dry and imaged on the EnderScope using the SnakeScan.py script. The filter paper was scanned in both reflected light and fluorescence mode (snake scan parameters: x_win = 17, y_win = 23, x_step = 6.2, y_step = 4.5. Reflected light scan: exposure = 10 000 000, wb.gain = 3, 1. Fluorescence scan: exposure = 10 000 000, wb.gain = 3, 1).

**Figure 7.**
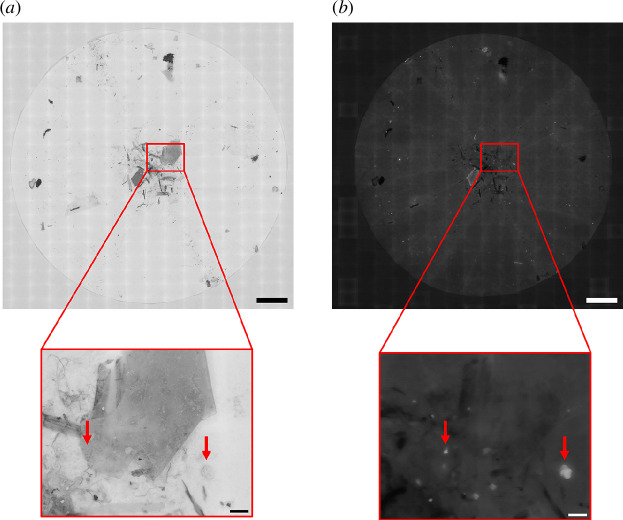
Composite image of Nile Red-stained MPs in environmental samples. Composite images of a filtered seawater sample that has been digested with KOH and stained with Nile Red. Images were acquired using the EnderScope in reflected light (*a*) and fluorescence (*b*) modes. The red callout box shows the same selected region of interest on each filter. The red arrows point to the same Nile Red-stained particles in each region of interest. Scale for composite images = 10 mm. Scale for selected regions of interest = 1 mm.

Following a similar protocol to 3.4.2, images were flipped horizontally and vertically. The red channel was extracted for fluorescence images, and a colour composite was generated for reflected light images. The fluorescence and reflected light images were both stitched into a large composite image with BigSticher (figure 7).

**Figure 8.**
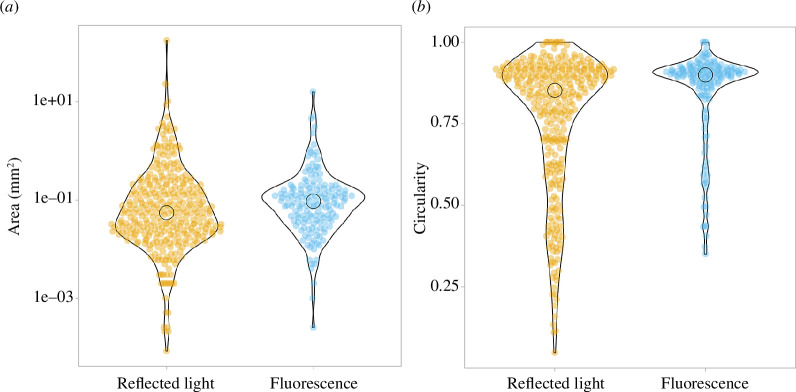
Area and circularity of particles detected in a seawater sample. A seawater sample was digested, filtered and stained with Nile Red (as described in §3.4.3). The filtered sample was imaged using the EnderScope in both reflected white light and fluorescence modes. Area (measured in mm²) of particles detected in the seawater sample is shown in (*a*) (reflected light: *n* = 329, median = 0.06, Q1 = 0.02, Q3 = 0.18; fluorescence: *n* = 154, median = 0.09, Q1 = 0.04, Q3 = 0.17). Circularity (measured from 0 to 1, 1 being a perfect circle) of detected particles is shown in (*b*) (reflected light: *n* = 329, median = 0.85, Q1 = 0.68, Q3 = 0.91; fluorescence: *n* = 154, median = 0.9, Q1 = 0.86, Q3 = 0.92).

It is clear that the digestion was not fully effective as some presumptive organic material is clearly visible on the filter paper in the reflected light scan. We see fluorescent particles including beads and fibres in the fluorescence scan (figure 7) that are not clearly distinguishable in the white light scan. The composite image was analysed by segmentation and particle detection as in §3.4.2, and the particle characteristics are detailed in figure 8.

## 4. Discussion

We have provided a detailed description of the EnderScope, along with examples of software and proof-of-concept experiments. The EnderScope is designed to be suitable for use inside and outside the formal research environment. For small-budget projects in established labs, the EnderScope is a low-cost yet effective tool for automated scanning of fluorescent and non-fluorescent samples. For members of the public, the EnderScope provides an opportunity to actively engage with science. As a hobbyist 3D printer, many members of the public may already own an Ender 3 Pro. With a small modification made from mostly 3D-printed parts, their 3D printer can be transformed into a scientific instrument. Individuals can then be empowered to ask questions about their local environment, particularly those related to MP pollution, and use the EnderScope to find answers.

While this benefits science as a whole, it is particularly valuable for addressing the issue of MP pollution. By enabling a larger number of people to measure MP pollution in different environments, the EnderScope can contribute to a more comprehensive understanding of the global MP pollution problem. The more measurements we have, the better we can establish a baseline for MP pollution, monitor its trends and implement measures to mitigate it.

This is not the first low-cost instrument designed with the intention of accelerating the detection of Nile Red-stained particles. For example, a smartphone-based imager has been developed by Leonard *et al*. [23] within a similar price range (500 USD). The novelty of the EnderScope is that it is essentially two tools in one: a 3D printer and a microscope. The use of a 3D printer in this design, while not only accounting for the mechanical components of this device, acts as a gateway into the world of fabrication. As the users successfully construct the EnderScope, they will have greater confidence to build more complex scientific instruments, and additionally the tools and knowledge to be able to do that.

By simplifying fluorescence microscopy down to its most basic form, we have created an instrument that is highly efficient in terms of its energy use. The transition from large, power-hungry instruments to many small, accessible tools not only lowers barriers to entry but also contributes to reducing the overall energy footprint of scientific research.

Detecting MPs using the Nile Red staining method, while cheaper and more accessible than spectroscopic methods, has several limitations. As Nile Red is a lipophilic dye, it may stain natural lipids that remain post-digestion. In addition, certain pigments, particle weathering and certain polymer types can quench the Nile Red fluorescence [24].

When digesting organic material in samples, the choice of digestion reagent will vary depending on several factors such as sample type and availability and cost of reagents. While KOH digestion was sufficient to show our instrument is effective, it may not be optimal for all applications. Users should ensure the chosen digestion method is appropriate for their own samples and applications. The relative merits of the different digestion methods are reviewed by Lusher *et al.* [5].

The EnderScope, as presented in this manuscript, is not in its final form. The design outlined above serves as an example to show the practicality and feasibility of the EnderScope concept. There are many aspects of the EnderScope design that can be improved upon. The design can be further refined to be simpler and include less parts. The GUI can be refined to be more user-friendly. The design could also be modified to become more elaborate. For example, using higher-magnification objective lenses, traditional C-Mount cameras for microscopy, traditional emission filters, dichroic mirrors and side-mount illumination. The NeoPixel LED ring can be used to achieve oblique illumination. If white light illumination is needed for true colour reproduction, the RGB LEDs can be replaced with slightly more expensive RGBW LEDs. Python scripts can be developed to improve particle detection, include LED control, perform automatic bed levelling with autofocus, run-time lapse imaging, etc. As the EnderScope is an open-source project, we hope it will evolve, adapt and improve over time through the collective efforts of the scientific community and the wider public [25].

## Data Availability

All raw data are available on Dryad [26]. Electronic supplementary material is available at [27].
